# N-Doped Graphene-Decorated NiCo Alloy Coupled with Mesoporous NiCoMoO Nano-sheet Heterojunction for Enhanced Water Electrolysis Activity at High Current Density

**DOI:** 10.1007/s40820-021-00607-5

**Published:** 2021-02-19

**Authors:** Guangfu Qian, Jinli Chen, Tianqi Yu, Lin Luo, Shibin Yin

**Affiliations:** College of Chemistry and Chemical Engineering, MOE Key Laboratory of New Processing Technology for Nonferrous Metals and Materials, State Key Laboratory of Processing for Non-Ferrous Metal and Featured Materials, Guangxi University, 100 Daxue Road, Nanning, 530004 P. R. China

**Keywords:** N-doped graphene-decorated NiCo alloy, Catalyst, Mesoporous nano-sheet, Water electrolysis, High current density

## Abstract

Developing highly effective and stable non-noble metal-based bifunctional catalyst working at high current density is an urgent issue for water electrolysis (WE). Herein, we prepare the N-doped graphene-decorated NiCo alloy coupled with mesoporous NiCoMoO nano-sheet grown on 3D nickel foam (NiCo@C-NiCoMoO/NF) for water splitting. NiCo@C-NiCoMoO/NF exhibits outstanding activity with low overpotentials for hydrogen and oxygen evolution reaction (HER: 39/266 mV; OER: 260/390 mV) at ± 10 and ± 1000 mA cm^−2^. More importantly, in 6.0 M KOH solution at 60 °C for WE, it only requires 1.90 V to reach 1000 mA cm^−2^ and shows excellent stability for 43 h, exhibiting the potential for actual application. The good performance can be assigned to N-doped graphene-decorated NiCo alloy and mesoporous NiCoMoO nano-sheet, which not only increase the intrinsic activity and expose abundant catalytic activity sites, but also enhance its chemical and mechanical stability. This work thus could provide a promising material for industrial hydrogen production.

**Supplementary Information:**

The online version contains supplementary material available at 10.1007/s40820-021-00607-5.

## Highlights

- N-doped graphene-coated structure and mesoporous nano-sheet can efficiently boost active sites and stability for hydrogen and oxygen evolution reaction.
- NiCo@C-NiCoMoO/NF exhibits low overpotentials for HER (266 mV) and OER (390 mV) at ± 1000 mA cm^−2^.
- For water electrolysis, it can hold at 1000 mA cm^−2^ for 43 h in 6.0 M KOH + 60 °C condition.

**Supplementary Information:**

The online version contains supplementary material available at 10.1007/s40820-021-00607-5.

## Graphical abstract

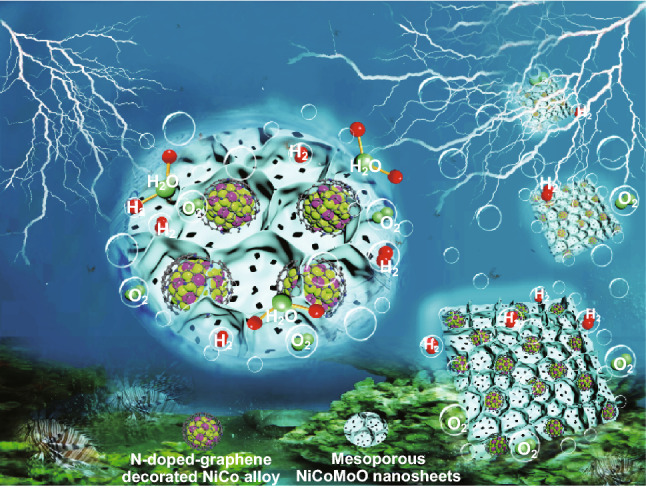

## Introduction

Water electrolysis (WE) can convert renewable sources (i.e., solar, wind) into H_2_ with clean and high energy density, but the sluggish kinetics of hydrogen and oxygen evolution reaction (HER and OER) at cathode and anode will hinder its efficiency [1–4]. Although Pt-/Ir-/Ru-based materials are the best choice to accelerate these two half-reactions, the large-scale hydrogen production is still limited by its shortage and high price [5–7]. Therefore, developing highly efficient non-precious metal materials to replace the noble metals for reducing cost and improving the performance of WE are necessary [8, 9].

Recently, 3D transition metal-based (TMB) catalysts are regarded as prospective alternative to noble metals, due to their abundance and low cost [10–14]. However, the 3D TMB catalysts are unstable under strong alkaline conditions. To address this problem, some researchers reported a novel strategy to construct the 3D TMB catalysts with N-doped graphene-encapsulated to improve the stability and catalytic activity [15]. Deng et al. prepared the ultrathin graphene layer encapsulating FeNi alloy and efficiently optimizing its surface electronic structure [16], and it obtains a low overpotential (280 mV) at 10 mA cm^−2^ for OER and can keep for 24 h. Mu et al. fabricated a Mo_2_C@C nanoball with hollow porous, which displayed low overpotentials for HER in 1.0 M KOH (115 mV) and 0.5 M H_2_SO_4_ (129 mV) solution at − 10 mA cm^−2^ [17]. Furthermore, other investigators also use the N-doped carbon-encapsulated 3D TMB catalysts, which can optimize the distribution of electrons on the metal surface and prevent metal dissolution under strong alkaline conditions to enhance the catalytic performance [18–20], while most of them are focusing on studying the catalytic performance at low current density and also need high potential to drive the WE. Therefore, it is deserved to develop 3D TMB materials with excellent WE catalytic activity at high current density [21–24].

Mesoporous-based materials are studied for enhancing the WE performance, because it has large specific surface area to expose abundant catalytic activity sites, increase the contact area with electrolyte, and prompt the gas and electrolyte diffusion at high current density [25–29]. Du et al. reported Co_4_N-CeO_2_ porous nano-sheet self-supported on graphite plate (Co_4_N-CeO_2_/GP), which shows low overpotentials for HER (24 mV) and OER (239 mV) at ± 10 mA cm^−2^ [30]. It can work at 500 mA cm^−2^ for 50 h as cathode and anode, exhibiting long-term durability. Ren et al. synthesized ternary 3D Ni_2(1−*x*)_Mo_2*x*_P nanowire with mesoporous structure; at − 500 and − 1000 mA cm^−2^, it exhibits low overpotentials for HER (240 and 294 mV) under 1.0 M KOH solution [31]. Although researchers synthesized many mesoporous materials with better electrocatalytic performance, the activity and durability at high current density still cannot meet the demand of industry WE. In addition, most of these catalysts are used only for HER or OER instead of overall water splitting.

In this work, we synthesize a highly efficient N-doped graphene-decorated NiCo alloy coupled with mesoporous NiCoMoO nano-sheet grown on 3D nickel foam as bifunctional catalyst (NiCo@C-NiCoMoO/NF) for WE. At ± 1000 mA cm^−2^, it exhibits excellent catalytic activity with low overpotentials for HER and OER (266 and 390 mV). More importantly, under 6.0 M KOH solution and 60 °C, it needs ultralow voltage of 1.90 V to reach 1000 mA cm^−2^ and can maintain for 43 h as anode and cathode.

## Material Synthesis and Characterization

### Synthesis of NiCo@C-NiCoMoO/NF Nano-Sheet

All reagents come from Aladdin Reagent Co., Ltd. without purification. First, nickel foam (NF, 2.0 × 4.0 cm^2^) was treated in ethanol, 3.0 M hydrochloric acid, and ultra-pure water with ultrasonication, respectively. Second, the cleaned NF was put in an 30 mL mix solution (ethylene glycol and ultra-pure water) with sodium molybdate dihydrate, urea, and nitrate hexahydrate. Third, the mix solution was put into steel autoclave for 12 h at 180 °C. After cooling to 25 °C, the NF was washed by ethanol and ultra-pure water, and dried overnight under vacuum at 80 °C. Finally, the dried sample was treated at 450 °C under 5% H_2_ + 95% Ar atmosphere for 2 h (3 °C min^−1^, the obtained sample named as NiCo@C-NiCoMoO/NF), and its mass load on NF was ≈ 10.5 mg cm^−2^. Besides, the dried sample was also heated at 350 and 550 °C. NiCo-NiCoMoO/NF nano-sheet was prepared in ultra-pure water with Ni, Co, and Mo source.

### Physical Characterization

The scanning electron microscopy (SEM, SU8220, HITACHI, Japan) was applied to research the surface morphology of the samples. X-ray diffraction (XRD) was examined on SmartLab, Rigaku Co., D8 Advance (Japan, *λ*_(Cu Kα)_ = 0.15406 nm). Transmission electron microscopy (TEM) and high-resolution TEM (HRTEM) and energy-dispersive X-ray (EDX) spectroscopy were characterized by a Titan ETEM G2 80-300 (FEI Co., USA). Raman spectroscopy was tested by a Raman spectrometer (Horiba Jobin Yvon Inc., France, *λ*_(He/Ne)_ = 532 nm). The N_2_ adsorption/desorption of the samples at 77 K was evaluated by the ASAP 2420 instrument (USA, Micrometrics Co.). The elements status of catalyst was tested by X-ray photoelectron spectroscopy (XPS, ESCALab 250Xi, Al X-ray, USA).

### Electrochemical Tests

All the electrochemical tests [linear sweep voltammetry (LSV), chronopotentiometry (CP), and electrochemical impedance spectroscopy (EIS)] used the standard three-electrode system [counter electrode: graphite bar; working electrode: the as-prepared samples (the test area is 0.5 cm^2^); reference electrode: reversible hydrogen electrode] by electrochemical workstation (Germany, ZAHNER) under 1.0 M KOH solution containing saturated N_2_. EIS was tested by the three-electrode system from 100,000 to 0.1 Hz; the test potential was − 0.2 and 1.5 V for HER and OER (the amplitude is 5 mV). The following formula was used for *iR* correction potential (*E*_corr_): (1) *E*_corr_ = *E*_mea_ − *iR*_s_, which was actually measured potential (*E*_mea_) and the solution resistance (*R*_s_). Besides, the same condition was used for two-electrode system. The equation [(2) *η* = *b*log|j|+ *a*] was used to assess the Tafel plots; the Tafel slope and the current density were *b* and *j*. The turnover frequency (TOF) and mass activity (MA) of catalyst for HER and OER were calculated based on the reported literatures [32–35].

Besides, 20 wt% Pt/C (anode) and 40 wt% IrO_2_/C (cathode) were used as noble metal ink (bought from Aladdin with no further treatment). Ethanol (0.96 mL) and 5.0 wt% Nafion (40.0 μL) mixed solution was applied to disperse this noble metal catalyst; then, it was dropped on NF (0.5 cm^2^) and named as Pt/C/NF and IrO_2_/C/NF.

## Result and Discussion

### Physicochemical Characterization

**Fig. 1:**
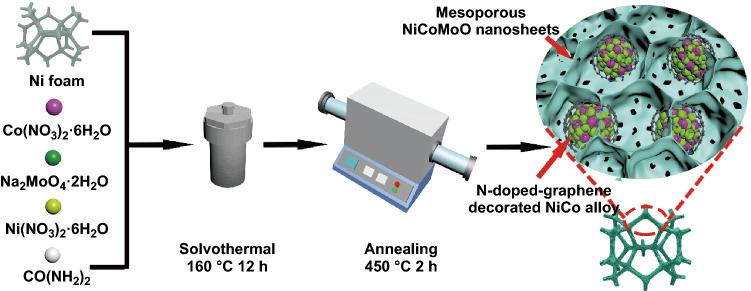
Schematic for the synthesis of NiCo@C-NiCoMoO/NF

N-doped graphene-decorated NiCo alloy coupled with mesoporous NiCoMoO nano-sheet grown on 3D nickel foam was synthesized via the facile two-step methods (Fig. 1).

Figure S1a, b displays the SEM images of NiCo@C-NiCoMoO/NF (annealed at 450 °C), which shows that the nanoparticles are uniformly anchored on the self-supported mesoporous nano-sheet, and it is different from the NiCoMoO nano-sheet precursors with smooth surfaces (Fig. S1c, d). The XRD images in Fig. S2 show that the three diffraction peaks belong to the (111), (200), and (220) planes of NiCo, respectively [36]. Furthermore, the other peaks can be assigned to Ni_2_Mo_3_O_8_ (PDF#37-0855) and Co_2_Mo_3_O_8_ (PDF#34-0511). The XRD results indicate that it composed of NiCo alloy, Ni_2_Mo_3_O_8_, and Co_2_Mo_3_O_8_.

**Fig. 2:**
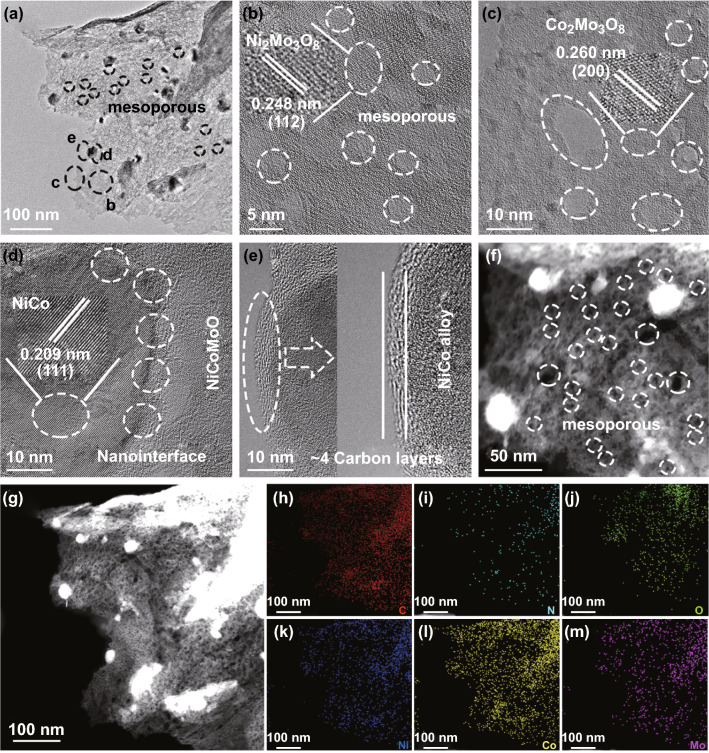
**a**–**e** TEM and HRTEM, **f**–**m** HAADF-STEM images and EDS elemental mappings of C, N, O, Ni, Co, and Mo of NiCo@C-NiCoMoO/NF

The TEM, high-angle annular dark field scanning TEM (HAADF-STEM), and high resolution TEM (HRTEM) images (Fig. 2a–g) are used to reveal the lattice fringe, N-doped graphene, mesoporous nano-sheet, and nanoparticles (≈ 30–50 nm) structure. The nanoparticles are evenly dispersed on the mesoporous nano-sheet (Fig. 2a). In Fig. 2b–d, the interplanar distances of 0.209 nm (111), 0.248 nm (112), and 0.260 nm (200) are assigned to NiCo, Ni_2_Mo_3_O_8_, and Co_2_Mo_3_O_8_ (consistent with XRD), respectively [37].

In Fig. 2d, the nanointerface existing between NiCo alloy and NiCoMoO can facilitate the redistribution of electrons to form the electron-rich and electron-poor species, which can optimize H* and H_2_O/OH^−^ absorption energy to enhance the performance for HER and OER [22, 38–40]. Furthermore, the NiCo alloy is obviously coated by graphene carbon (~ 4 layers) in Fig. 2e, which can efficiently optimize the distribution of electrons on the catalyst’s surface to improve the catalytic activity for WE and prevent the metal dissolution under strong alkaline condition [41].

The graphene carbon of NiCo@C-NiCoMoO/NF is also evaluated by Raman in Fig. S3, and the ratio of area D and G is 1.36 at 450 °C, which is larger than the ones prepared at 350 and 550 °C (1.16 and 1.28), suggesting a larger number of structural defects to enhance the catalytic activity for WE. The EDS elemental mappings (Fig. 2h–m) demonstrate that the Ni, Co, Mo, O, C, and N elements are evenly distributed on NiCo@C-NiCoMoO/NF nano-sheet.

Furthermore, it also exhibits mesoporous structure (2–15 nm) in Figs. 2b, c and S4a–c, which can be obtained by HAADF-STEM (Figs. 2f and S4d, e). To further study the mesoporous structure of NiCo@C-NiCoMoO/NF nano-sheet, the pore volume/size (0.18 cm^3^ g^−1^/6.83 nm) and specific surface area (102.96 m^2^ g^−1^) are characterized by N_2_ absorption/desorption measurements, and the most parts range of mesoporous peaks is 1.0–14.0 nm (Fig. S5a, b and Table S1). This mesoporous nano-sheet possesses a large specific surface area to expose more catalytic active sites and enhance the activity for WE. Additionally, it can increase the contact area with electrolyte to accelerate the release of H_2_/O_2_ bubbles and improve the performance for WE at high current density [26, 28].

Meanwhile, the NiCoMoO nano-sheet precursors also annealed at 350 and 550 °C to study the effect of post-treatment at different temperatures on the crystal structure, morphology, pore volume/size, and specific surface area (Fig. S5 and Table S1). When the precursors annealed at 350 °C, the XRD peak intensities of Co_2_Mo_3_O_8_, Ni_2_Mo_3_O_8_, and NiCo are too weak (Fig. S2), the nano-sheets are smooth (Fig. S6a, b), and the size of mesoporous is mainly concentrated on 11.1 nm (Fig. S5c, d). As annealed at 550 °C, the XRD peak intensities of Co_2_Mo_3_O_8_, Ni_2_Mo_3_O_8_, and NiCo are strong, the nano-sheets are broken (Fig. S6c, d), and the material has macroporous structure (Fig. S5e, f). Thus, temperature plays an important effect on the formation of this novel structure.

**Fig. 3:**
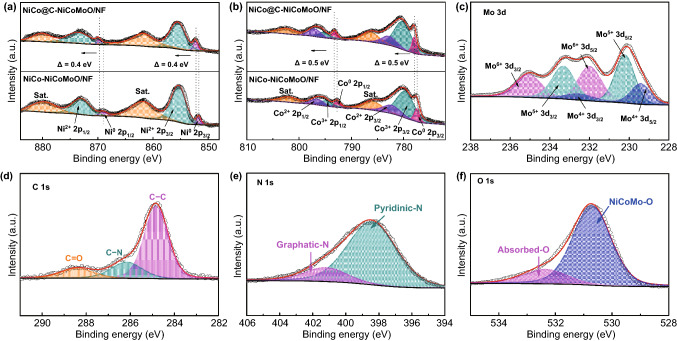
**a**, **b** High-resolution XPS spectra of Ni 2*p* and Co 2*p* for NiCo@C-NiCoMoO/NF and NiCo-NiCoMoO/NF. **c** Mo 3*d*, **d** C 1*s*, **e** N 1*s*, and **f** O 1*s* of NiCo@C-NiCoMoO/NF

Subsequently, the electron interaction and elemental status of Ni, Co, Mo, O, C, and N elements in NiCo@C-NiCoMoO/NF (Figs. 3 and S7) are proved by XPS. Interestingly, for NiCo@C-NiCoMoO/NF, the Ni 2*p* peaks of Ni show a ≈ 0.4 eV positive shift compared with that of NiCo-NiCoMoO/NF (Fig. 3a). For Co 2*p*, the peaks of NiCo@C-NiCoMoO/NF also show a ≈ 0.5 eV positive shift as against to that of NiCo-NiCoMoO/NF (Fig. 3b). This is because of different electronegativity between Ni/Co (1.91/1.88), C (2.55), and N (3.04). So, the N-doped carbon can efficiently optimize the electron structure on the surface of NiCo alloy, which could be beneficial to enhance the performance for WE.

Particularly, the redistribution of electrons could lead to the charge transfer from NiCo to N-doped carbon, forming electron-rich N-doped graphene and electron-poor NiCo species, which can optimize the absorption energy of H*, H_2_O, and OH^−^ for HER and OER [38, 39]. The high-resolution XPS (HRXPS) spectra of Mo 3d are fitted into six mainly peaks at Mo^6+^ (235.1/232.0), Mo^5+^ (233.3/230.2), and Mo^4+^ (232.5/229.4), respectively (Fig. 3c). Besides, the surface of catalyst is oxidized when exposed to air, resulting in high valence state of Ni, Co, and Mo (Fig. 3a–c). As shown in Fig. 3d, the C 1*s* has three peaks at C=O (288.4 eV), C–N (286.2 eV), and C–C (284.8 eV) that further prove the existence of the N-doped graphene. Moreover, the N 1*s* is located at 401.3 and 398.5 eV in Fig. 3e, assigned to graphitic-N and pyridinic-N, which can result in an important effect on catalytic activity for HER and OER [42]. The peaks of O 1*s* are absorbed-O (532.4 eV) and NiCoMo-O (530.7 eV) in Fig. 3f, which demonstrate the exist of Ni_2_Mo_3_O_8_ and Co_2_Mo_3_O_8_.

Therefore, we can draw the concision that the N-doped graphene-decorated NiCo alloy coupled with mesoporous NiCoMoO nano-sheet  is successfully prepared. Combining SEM, BET, XRD, and Raman (Figs. S1–S6) results, the following formation mechanism can be proposed: different adsorption enthalpies of Ni, Co, and Mo can lead to the part of Ni and Co atoms segregated from precursors to form NiCo alloy [43, 44]. When the precursors annealed at 450 °C, parts of Ni and Co combine with O and Mo to form Ni_2_Mo_3_O_8_ and Co_2_Mo_3_O_8_, which can form the heterojunction between NiCo and NiCoMoO [37]. Besides, NiCo alloy can catalyze the organic carbon to form N-doped graphene [45]. The formation of mesoporous is caused by the dehydration from the precursors during the high-temperature calcination process, and the pore size is related to the temperature. However, when the precursors are annealed at 350 °C, the NiCo alloy cannot be reduced from the precursors, and the surface organic carbon cannot form more N-doped graphene. This will decrease the intrinsic activity for WE. It can also be seen from SEM and BET pictures (Figs. S6a, b and S5c, d), the material cannot be dehydrated to form a mesoporous nano-sheet structure due to the low temperature that cannot provide enough specific surface area for exposing more active sites. When the precursors annealing at 550 °C, the Ni and Co atoms will be quickly reduced to form strong NiCo alloy and NiCoMoO (Fig. S2), and the nano-sheet is quickly dehydrated to form the macroporous structure and almost broken as nanoparticles (Fig. S6c, d). It will lower the intrinsic activity for WE and not provide large specific surface area for exposing more active sites. In summary, the N-doped graphene-decorated NiCo alloy, mesoporous NiCoMoO nano-sheet, and heterostructures are formed at 450 °C, which have the highest intrinsic activity and specific surface area. The heterostructures owe good electrochemical activity for HER and OER that is confirmed by LSV, Tafel, and EIS characterization (Figs. S11 and S22).

### HER Catalytic Performance of NiCo@C-NiCoMoO/NF

**Fig. 4:**
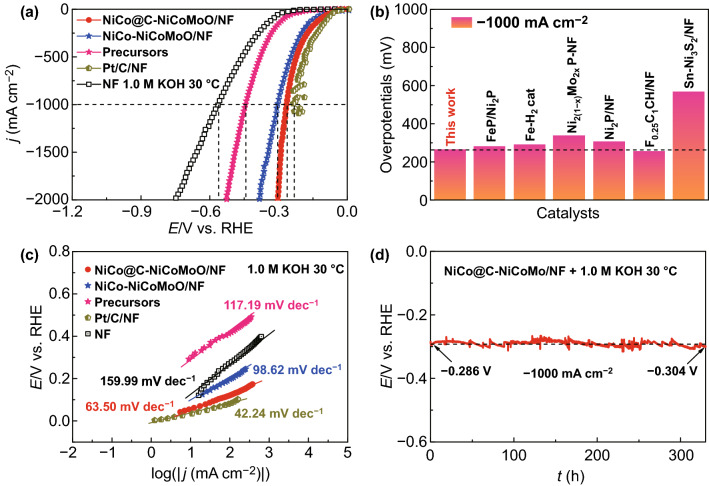
**a** LSV curves of HER for investigated samples. **b** Comparisons of HER overpotentials in this work and reported catalysts [31, 47–52]. **c** Corresponding Tafel curves. **d** CP curve

The HER electrocatalytic activity of the samples is evaluated by a three-electrode system under 1.0 M KOH solution containing saturated N_2_. Obviously, the NiCo@C-NiCoMoO/NF only acquires low overpotentials of 39 and 266 mV at − 10 and − 1000 mA cm^**−**2^ (Figs. 4a and S8), which is lower than that of NiCo-NiCoMoO/NF (*η*_−10_ = 75 mV; *η*_−1000_ = 303 mV). Thus, the HER activity of NiCo@C-NiCoMoO/NF is significantly improved after the NiCo alloy coated by N-doped graphene, especially at high current density, which could be attributed to the N-doped graphene structure, optimized the surface electronic distribution, and improved the activity and conductivity of catalysts. Furthermore, the overpotentials of NiCo@C-NiCoMoO/NF are lower than that of precursors (*η*_−10_ = 141 mV; *η*_−1000_ = 443 mV), NF (*η*_−10_ = 223 mV; *η*_−1000_ = 561 mV) and closed to Pt/C/NF (*η*_−10_ = 30 mV; *η*_−1000_ = 231 mV). The overpotential at − 1000 mA cm^−2^ is better than most of the reported literatures (Fig. 4b), which indicates that it could meet the demand of catalytic activity at high current density for industrial-scale.

Figure S9 displays the LSV curves of NiCo@C-NiCoMoO/NF with/without *iR* correction for HER. Tafel slope obtained from LSV curve is carried out to further research the kinetic of HER (Fig. 4c). It shows that Tafel slope of NiCo@C-NiCoMoO/NF is only 63.50 mV dec^−1^, outperforming NiCo-NiCoMoO/NF (98.62 mV dec^−1^), precursors (117.19 mV dec^−1^), NF (159.99 mV dec^−1^), and similar with Pt/C/NF (42.24 mV dec^−1^). The smaller value of Tafel suggests that NiCo@C-NiCoMoO/NF can readily overcome the kinetics process of HER. As shown in Table S3, TOF and MA values of NiCo@C-NiCoMoO/NF at the overpotentials of 50, 100, 150, and 200 mV also indicate its high catalytic activity for HER and better than most reported results in the literatures (Table S4).

The EIS is used to estimate the kinetics of HER. In Fig. S10, it displays that NiCo@C-NiCoMoO/NF has the smallest charge transfer resistance (*R*_ct_) compared with another samples, revealing its best electron transfer rate. For precursors annealed at different temperatures, the LSV curves, Tafel slope, and EIS of HER are displayed in Fig. S11, which demonstrate that the precursors annealing at 450 °C exhibits the best activity.

The electrochemical active surface area (EASA) is evaluated by the double-layer capacitance (*C*_dl_) that is obtained by cyclic voltammetry (CV) methods under no Faradic regions (Fig. S12). NiCo@C-NiCoMoO/NF has the largest *C*_dl_ value (28.81 mF cm^−2^); it is better than NiCo-NiCoMoO/NF (17.60 mF cm^−2^), indicating that N-doped carbon-decorated NiCo alloy can effectively boost the intrinsic activity and speed up the HER process. The LSV curves are normalized by EASA in Fig. S13; apparently, the intrinsic catalytic activity of NiCo@C-NiCoMoO/NF is better than NiCo-NiCoMoO/NF.

In Fig. 4d, we research the HER durability of NiCo@C-NiCoMoO/NF under 1.0 M KOH solution by CP measurement at − 1000 mA cm^−2^, which displays excellent stability after continuous work 340 h, and the potential has only changed 18 mV. Furthermore, the value of overpotential at − 1000 mA cm^−2^ and *R*_ct_ at − 0.2 V (*vs* RHE) after stability test is negligibly changed, indicating its good stability. The SEM (Fig. S15) and HRTEM (Fig. S16) images of NiCo@C-NiCoMoO/NF after HER stability test showed that it maintains the pristine morphology. In addition, the HRXPS (Fig. S17) spectra of Mo, Ni, and Co for NiCo@C-NiCoMoO/NF show no obvious change. These results confirm that the NiCo@C-NiCoMoO/NF exhibits an outstanding HER durability in 1.0 M KOH solution. The reason could be due to that the N-doped graphene-decorated NiCo alloy framework can prevent metal from dissolving in the strong alkaline solution, thus improving the chemical stability. Furthermore, the self-supporting mesoporous nano-sheet has large specific surface area to increase wettability of the catalyst, facilitate the release of H_2_ bubbles, avoid the use of binder to improve the electron transfer efficiency, and prevent the active substance from spalling to enhance the mechanical stability.

### OER Catalytic Performance of NiCo@C-NiCoMoO/NF

**Fig. 5:**
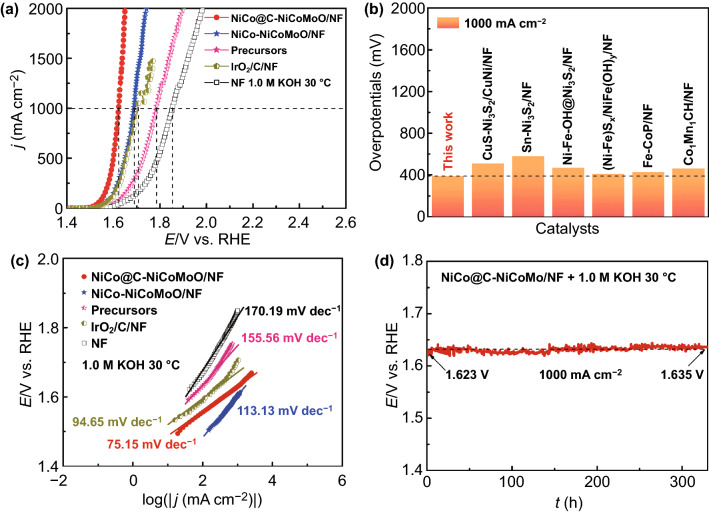
**a** LSV curves of OER for investigated samples. **b** Comparisons of OER overpotentials in this work and reported catalysts [8, 47, 50, 53–56]. **c** Corresponding Tafel curves. **d** CP curve

We evaluate OER catalytic performance of NiCo@C-NiCoMoO/NF under the same solution. Figures 5a and S18 show their *iR* corrected LSV curves. Similar to HER performance, NiCo@C-NiCoMoO/NF has the low overpotentials (260 and 390 mV) at 10 and 1000 mA cm^−2^, which is smaller than that of NiCo-NiCoMoO/NF (280 and 459 mV), indicating that the N-doped graphene can efficiently adjust the surface electronic of the catalyst to enhance the intrinsic activity of catalyst. In addition, it outperforms precursors (*η*_10_ = 320 mV; *η*_1000_ = 554 mV), IrO_2_/C/NF (*η*_10_ = 290 mV; *η*_1000_ = 476 mV), and NF (*η*_10_ = 340 mV; *η*_1000_ = 624 mV). Importantly, the overpotential at 1000 mA cm^−2^ is better than most of the reported literatures as shown in Fig. 5b. Furthermore, the LSV curves of OER for NiCo@C-NiCoMoO/NF with/without *iR* correction are shown in Fig. S19, and the LSV curves of OER for NiCo@C-NiCoMoO/NF and NiCo-NiCoMoO/NF are normalized by EASAs in Fig. S20 similar to HER performance.

We obtain the Tafel slopes from the LSV curves to evaluate the kinetics of OER (Fig. 5c). Obviously, the Tafel slopes are decreased in following order: NF (170.19 mV dec^−1^) > precursors (155.56 mV dec^−1^) > NiCo-NiCoMoO/NF (113.13 mV dec^−1^) > IrO_2_/C/NF (94.65 mV dec^−1^) > NiCo@C-NiCoMoO/NF (75.15 mV dec^−1^). The EIS is used to estimate the electrode kinetics of OER. Figure S21 displays that NiCo@C-NiCoMoO/NF has the smallest *R*_ct_ compared with another samples, revealing it possesses the best electron transfer rate. The above results also suggest that N-doped graphene-decorated NiCo alloy coupled with mesoporous NiCoMoO nano-sheet can effectively speed up the OER process. Additionally, in Fig. S22, the catalytic activity is different when precursors annealed at 350, 450, and 550 °C, and the precursors reducing at 450 °C exhibit the best activity. Subsequently, the calculated TOF and MA values of NiCo@C-NiCoMoO/NF for OER (Table S5) display its fast OER kinetics, which is higher than most reported literatures (Table S6).

The stability is also essential to evaluate the performance of catalyst, especially at high current density. As shown in Fig. 5d, NiCo@C-NiCoMoO/NF can keep for 340 h at 1000 mA cm^−2^, and the change of potential is 12 mV, displaying an outstanding stability. Furthermore, we also study the catalytic activity after stability test by LSV and EIS curves (Fig. S23), and it shows ignorable change. The outstanding durability of NiCo@C-NiCoMoO/NF could be assigned to the N-doped graphene-decorated NiCo alloy, which can avoid corrosion in the harsh alkaline environment, thus improving the chemical stability. Besides, the self-supporting mesoporous nano-sheet can enhance the mechanical stability, since it has large specific surface area to increase the contact area with electrolyte and prompt the release of O_2_ bubbles.

After the durability tests, SEM images of NiCo@C-NiCoMoO/NF maintain the pristine morphology (Fig. S24), and HRTEM images show that the mesoporous nano-sheet structure keeps well (Fig. S25), suggesting its excellent stability. In addition, the peak of the Ni^0^ and Co^0^ disappeared (Fig. S26a, b), which indicates that the surface of catalyst is oxidized during the OER process, and it could form the Ni/CoOOH [46]. As displayed in Fig. S26c, the XPS spectra of Mo^4+^ are also oxidized to Mo^6+^ and Mo^5+^, further suggesting the surface oxidation.

### WE Catalytic Performance of NiCo@C-NiCoMoO/NF

**Fig. 6:**
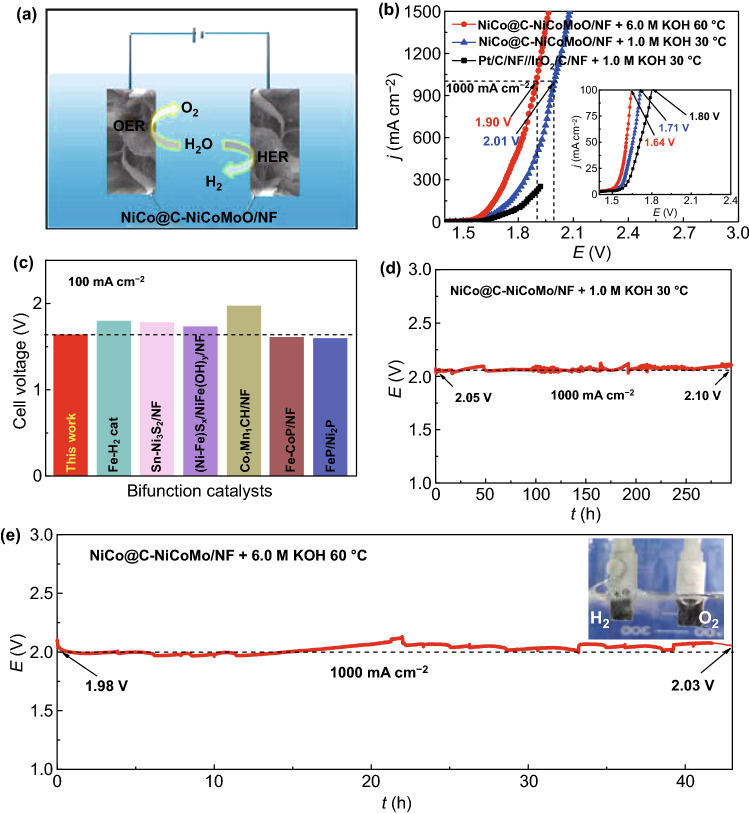
**a** Using NiCo@C-NiCoMoO/NF as the cathode and anode for water electrolysis. **b** LSV curves of OER for the investigated samples. **c** Comparison WE potentials for this work and reported catalysts [8, 47, 51, 53, 55, 57]. **d**, **e** CP curves of NiCo@C-NiCoMoO/NF under 1.0 M KOH + 30 °C and 6.0 M KOH + 60 °C

Based on the excellent performance of NiCo@C-NiCoMoO/NF toward HER and OER in alkaline solution, the two-electrode system is used to evaluate the WE performance by using it as bifunctional catalyst (Fig. 6a).

In Fig. 6b, at 100 mA cm^−2^, under 1.0 M KOH solution at 30 °C, WE performance of NiCo@C-NiCoMoO/NF (1.71 V) is better than that of the Pt/C/NF‖IrO_2_/C/NF (1.80 V) couple; it is smaller than most of the reported datum as shown in Fig. 6c. Interestingly, the NiCo@C-NiCoMoO/NF only requires a low potential of 2.01 V to deliver 1000 mA cm^−2^ and perform for 295 h with negligible change (50 mV, Fig. 6d), indicating that it is promising for industrial hydrogen production.

Additionally, as exhibited in Fig. S27, the amount of H_2_ and O_2_ is acquired by water drainage method at 0, 25, 50, 75, 100, and 125 min operating at ± 10.0 mA. Figure S27a, b shows the volume ratio of H_2_ and O_2_ is about 2:1, which is consistent with theoretical values, suggesting the closely 100% Faradic efficiency for WE.

Subsequently, NiCo@C-NiCoMoO/NF is tested in 6.0 M KOH + 60 °C (Fig. 6b, e); it only needs 1.90 V at 1000 mA cm^−2^ and can keep for 43 h without obvious attenuation. Therefore, NiCo@C-NiCoMoO/NF with excellent performance provides a promising material for WE to hydrogen production.

## Conclusions

In summary, NiCo@C-NiCoMoO/NF, a unique N-doped graphene-encapsulated structure and self-supported mesoporous nano-sheet, is prepared by solvothermal method and annealing treatment. As bifunctional catalyst, it displays outstanding HER and OER performance in 1.0 M KOH solution, which only needs overpotentials of 266 and 390 mV at ± 1000 mA cm^−2^, and shows superior stability for 340 h with no evident activity decrease. More importantly, when applied as anode and cathode in 6.0 M KOH + 60 °C, it exhibits a low potential of 1.90 V at 1000 mA cm^−2^ and can work for 43 h without obvious attenuation, exhibiting performance close to actual application. Therefore, this work may provide a promising catalyst with high catalytic activity and stability for industrial water electrolysis.

## Supplementary Information

Below is the link to the electronic supplementary material.

Supplementary file 1 (PDF 3588 KB)
