# Supplementary material for: N-Doped Graphene-Decorated NiCo Alloy Coupled with Mesoporous NiCoMoO Nano-sheet Heterojunction for Enhanced Water Electrolysis Activity at High Current Density
Source: Nanomicro Lett. 2021 Feb 19;13:77. doi: 10.1007/s40820-021-00607-5 (PMC8187493; doi:10.1007/s40820-021-00607-5)
Supplement: Supplementary file 1 — (PDF 3588 KB) [file 40820_2021_607_MOESM1_ESM.pdf]

Supporting Information for

N-Doped-Graphene Decorated NiCo Alloy Couple with Mesoporous NiCoMoO Nano-sheets Heterojunction for Enhanced Water Electrolysis Activity at High Current Density

Guangfu Qian[1], Jinli Chen[1], Tianqi Yu[1], Lin Luo[1], Shibin Yin[1,]*

[1]College of Chemistry and Chemical Engineering, MOE Key Laboratory of New Processing Technology for Nonferrous Metals and Materials, State Key Laboratory of Processing for Non-Ferrous Metal and Featured Materials, Guangxi University, 100 Daxue Road, Nanning 530004, P. R. China

*Corresponding author. E-mail: yinshibin@gxu.edu.cn (S. Yin)

Supplementary Tables and Figures

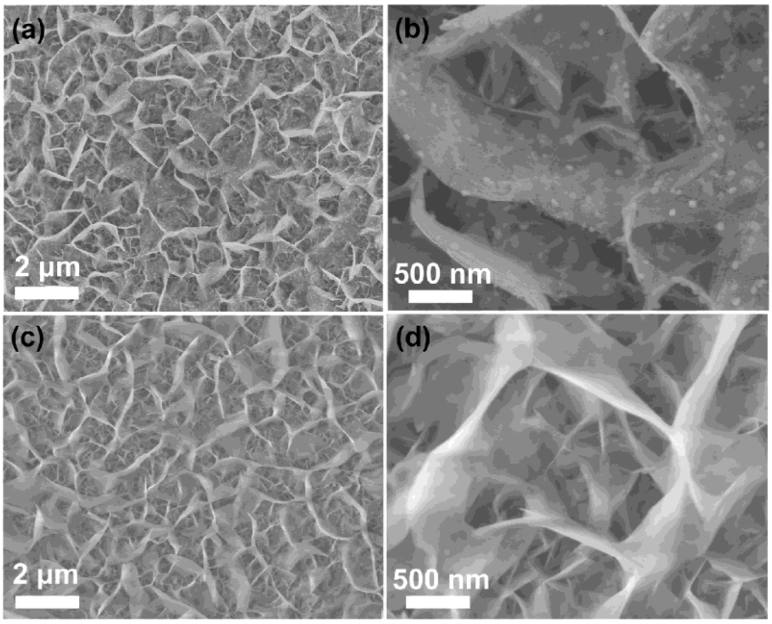

Fig. S1 SEM images of (**a**, **b**) precursors annealed at 450 °C and (**c**, **d**) precursors

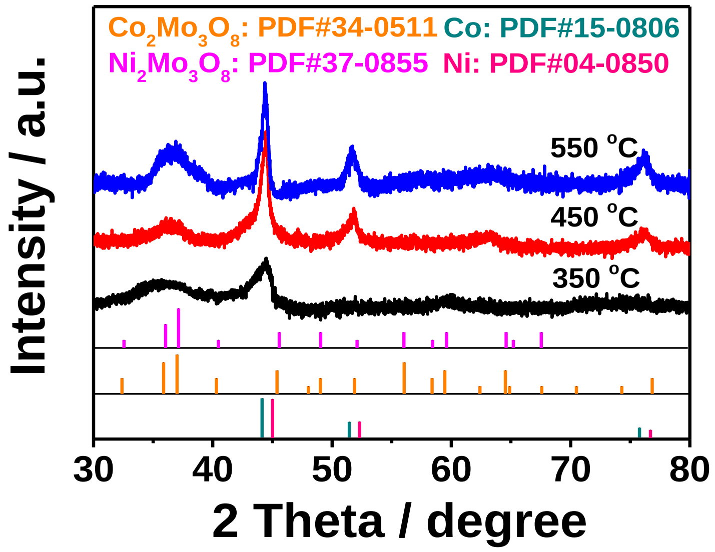

Fig. S2 XRD spectra of precursors annealed at different temperatures

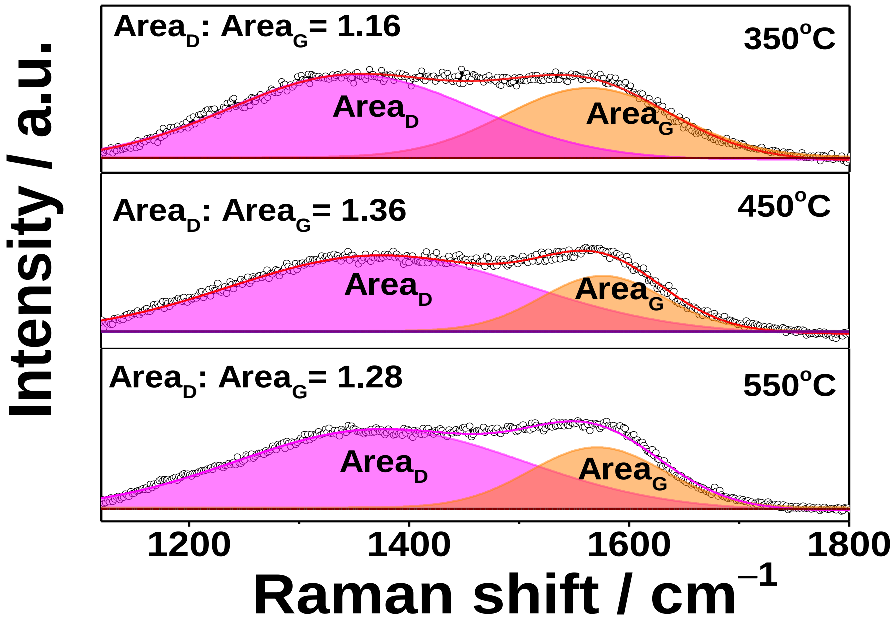

Fig. S3 Raman spectra of precursors annealed at different temperatures

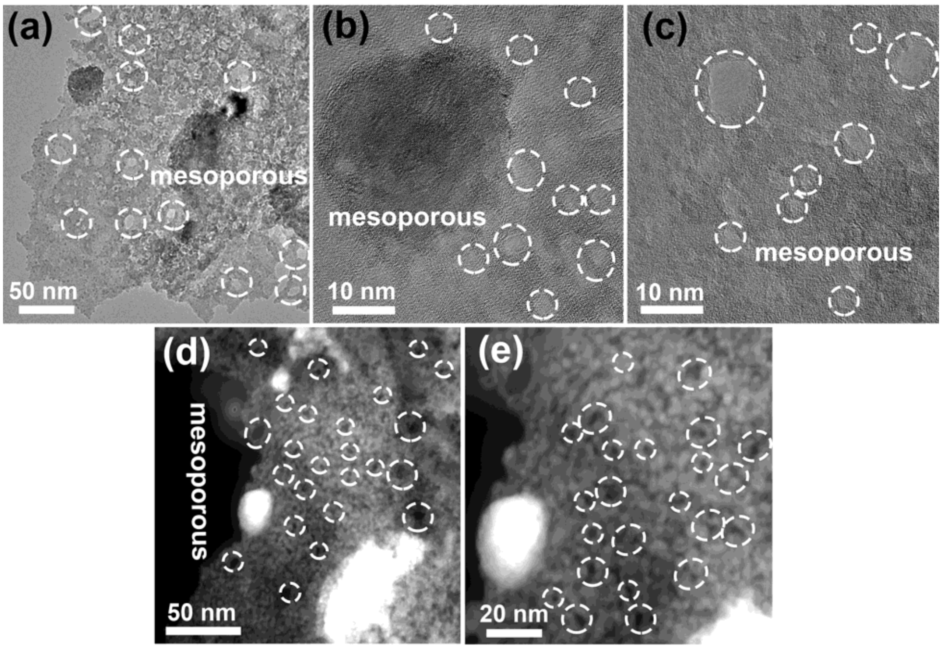

Fig. S4 (**a-c**) TEM and HRTEM and (d, e) HAADF-STEM images of NiCo@C-NiCoMoO/NF

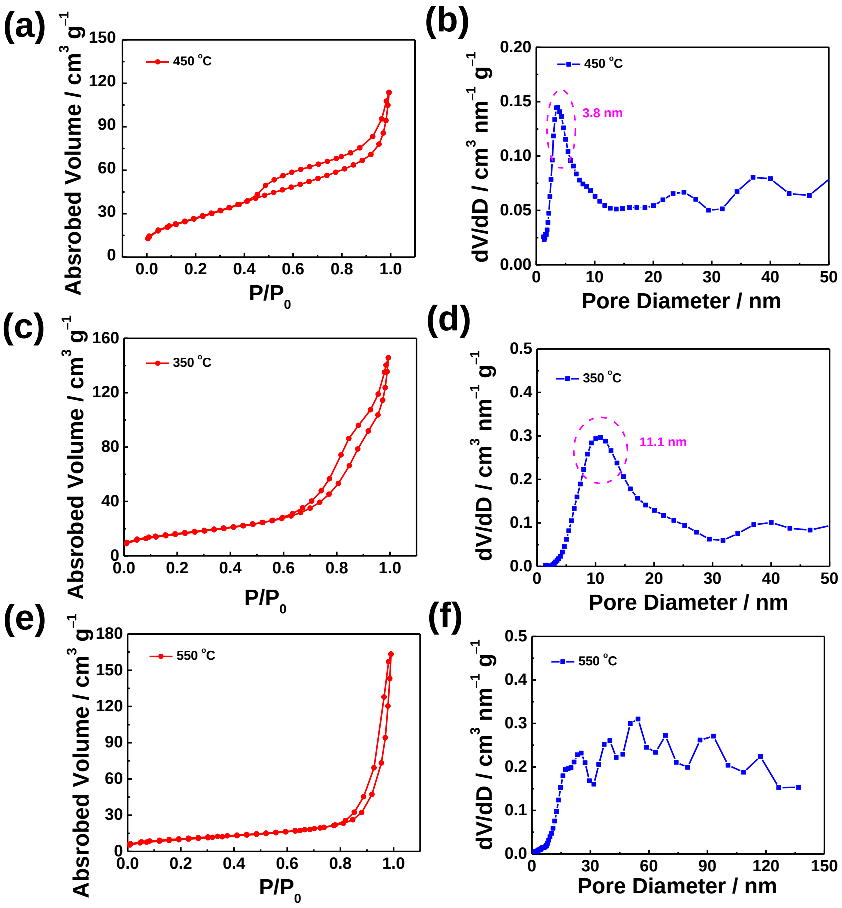

Fig. S5 (**a, c, e**) N_2 adsorption/desorption isotherms and (**b, d, f**) the corresponding pore size distributions of precursors annealed at different temperatures

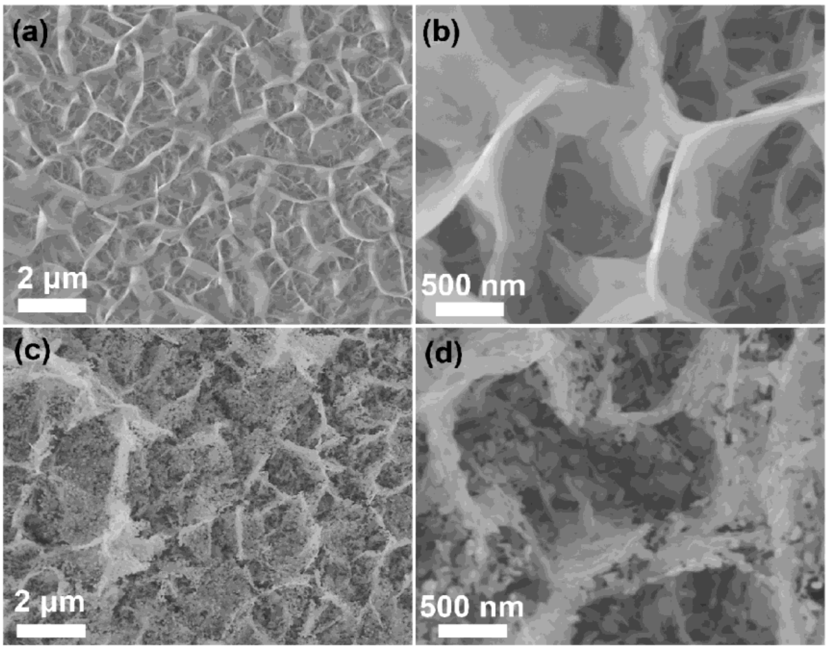

Fig. S6 SEM images of precursors annealed at (**a, b**) 350 °C and (**c, d**) 550 °C

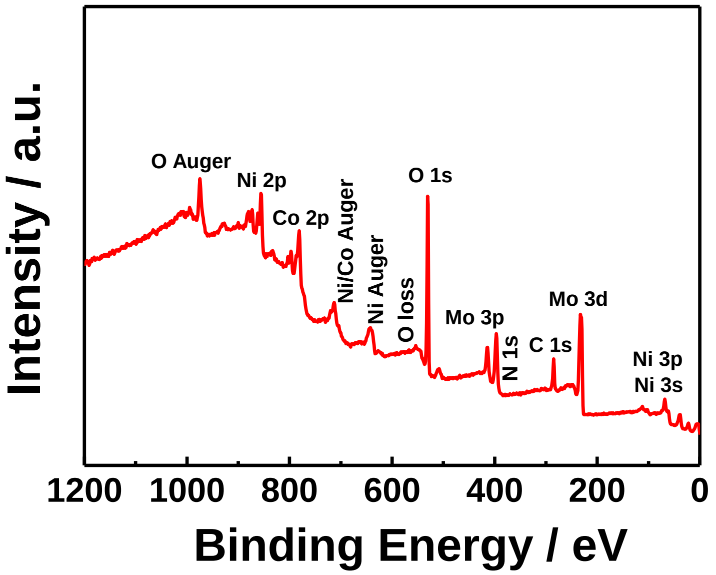

Fig. S7 XPS summary spectra of NiCo@C-NiCoMoO/NF

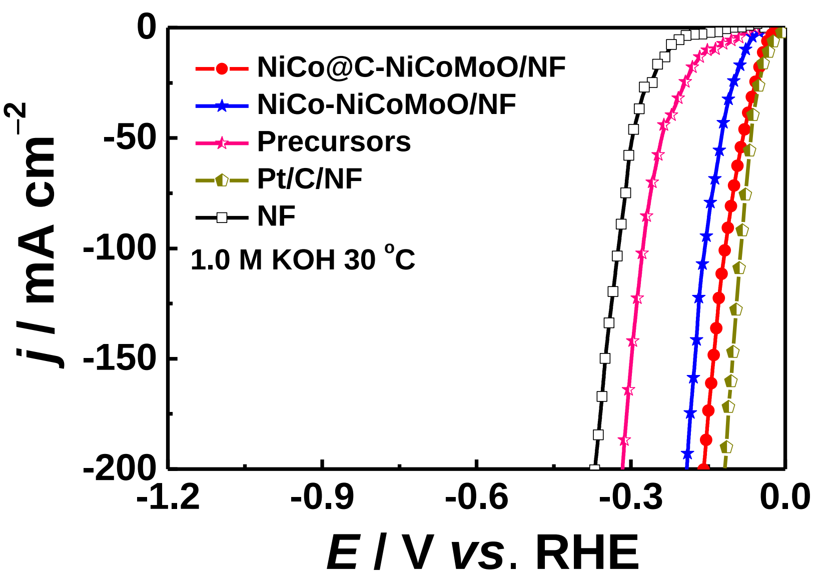

Fig. S8 LSV curves of HER for investigated samples

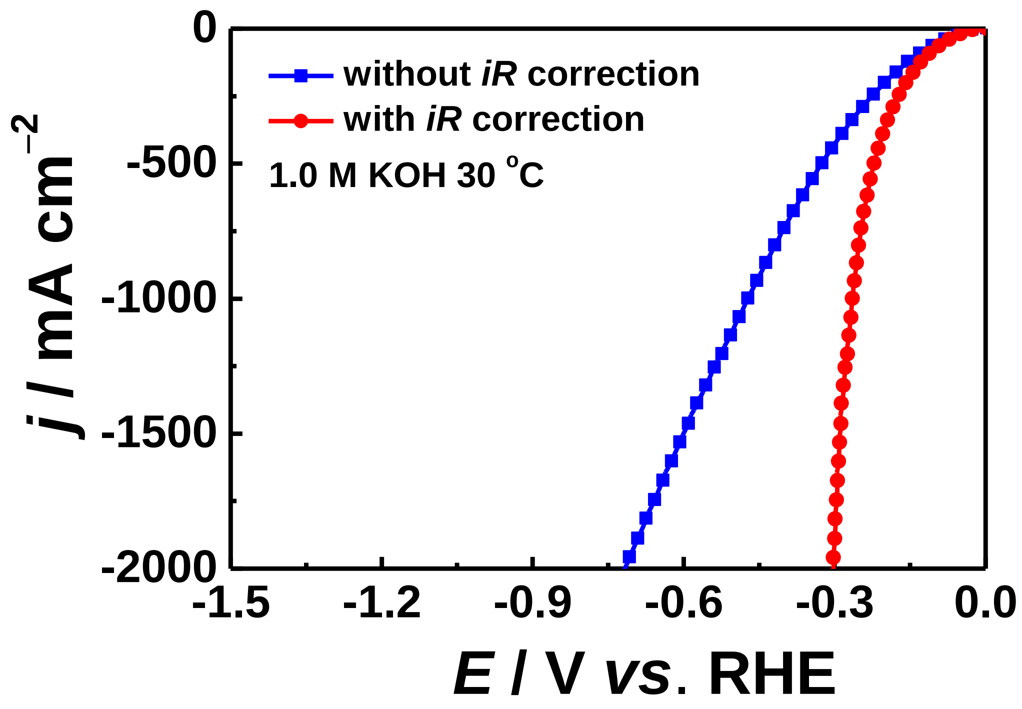

Fig. S9 LSV curves of NiCo@C-NiCoMoO/NF with/without *iR* correction

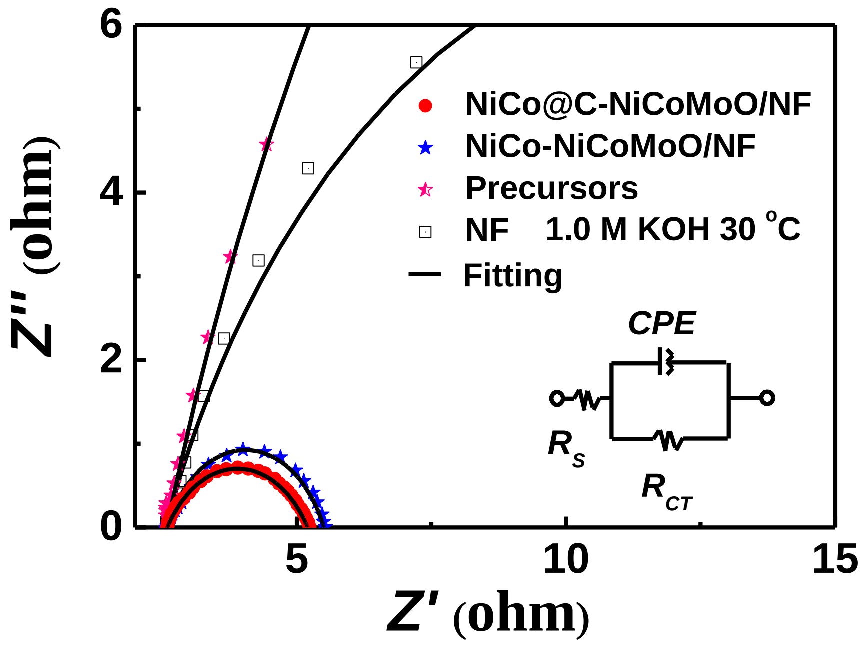

Fig. S10 Nyquist plots tested at −0.2 V for HER with a frequency from 100,000 to 0.1 Hz in 1.0 M KOH; inset is the equivalent circuit model

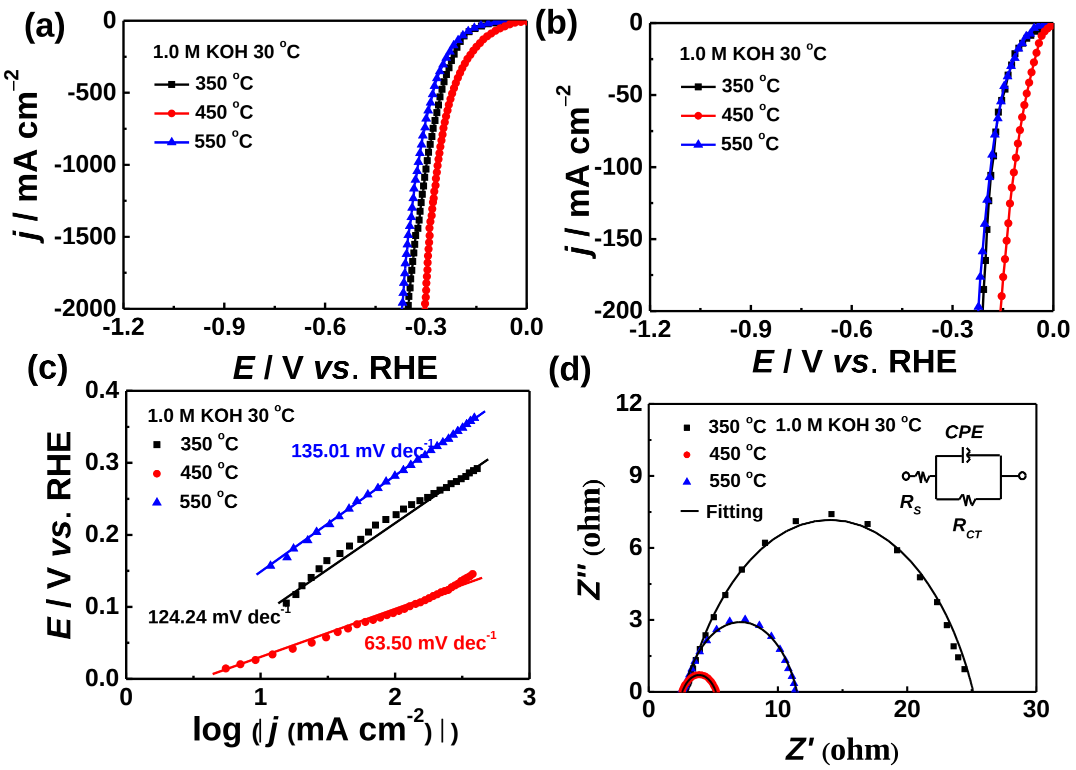

Fig. S11 (**a-d**) LSV curves of HER for precursors annealed at different temperatures

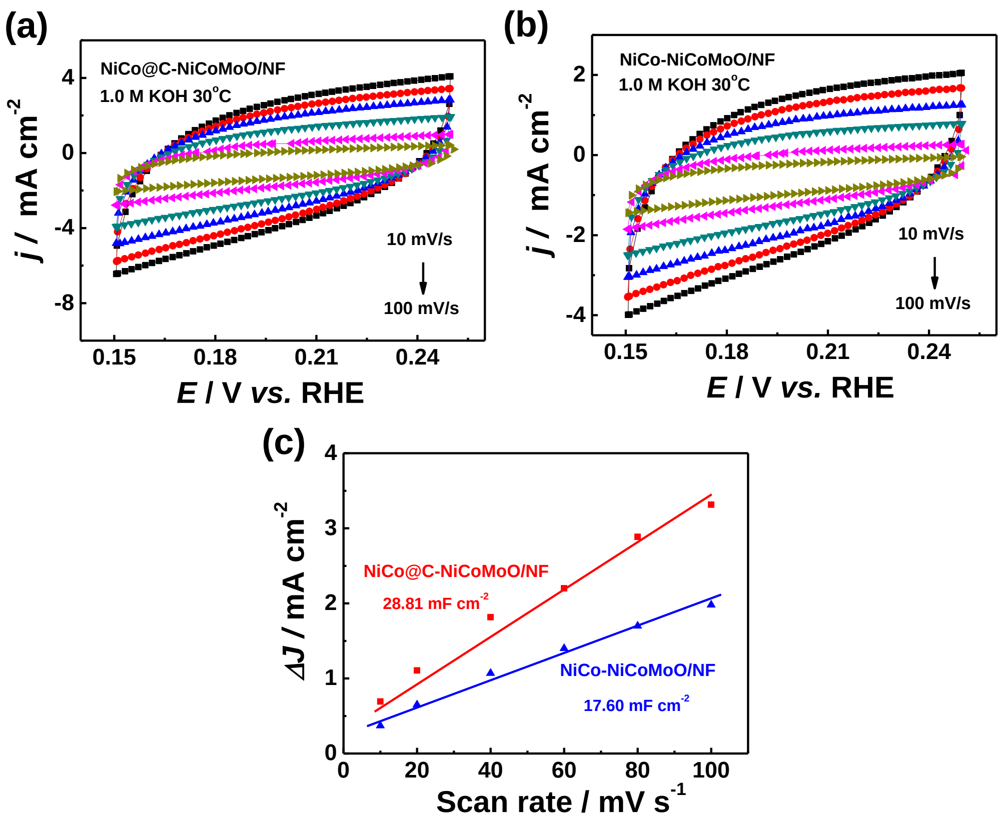

Fig. S12 (**a, b**) Typical CVs of the samples with scan rates ranging from 10 to 100 mV s^{-1}, the scanning potential range is from 0.15 V to 0.25 V; (**c**) Estimation of C_{dl} by plotting the capacitive current density against the scan rate to fit a linear regression

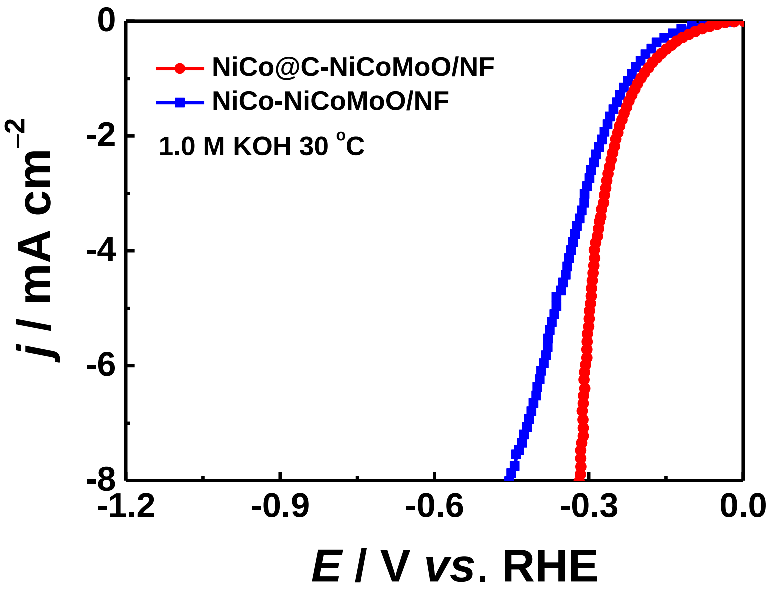

Fig. S13 LSV curves of HER for NiCo@C-NiCoMoO/NF and NiCo-NiCoMoO/NF normalized by EASAs

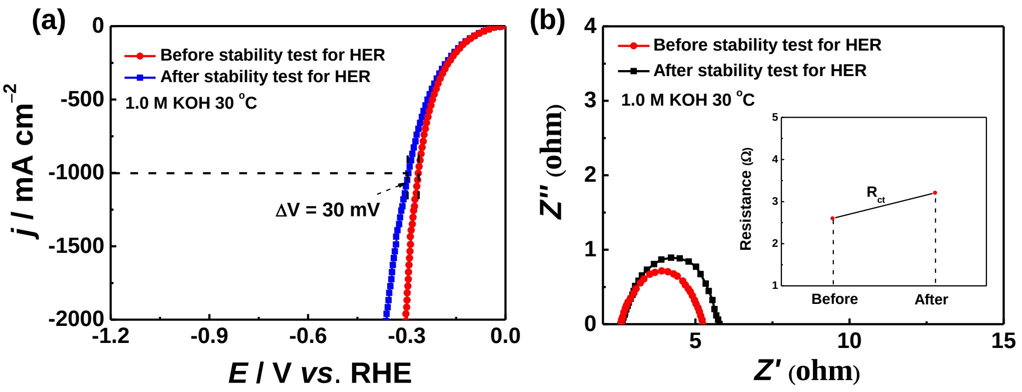

Fig. S14. (**a**) LSV curves and (**b**) R_{ct} of NiCo@C-NiCoMoO/NF before and after HER stability test

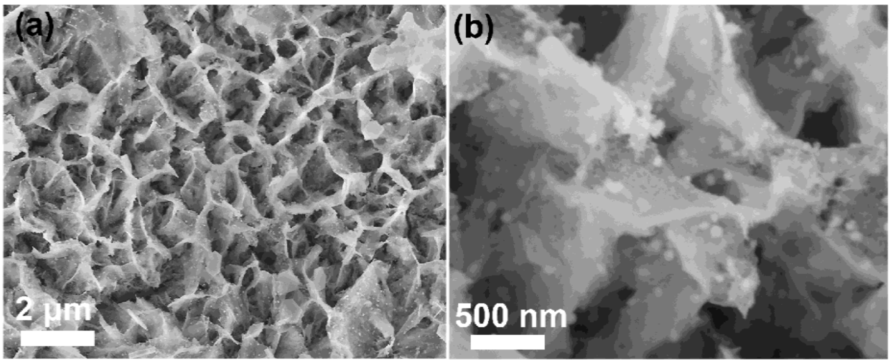

Fig. S15 (**a, b**) SEM images of NiCo@C-NiCoMoO/NF after HER stability test

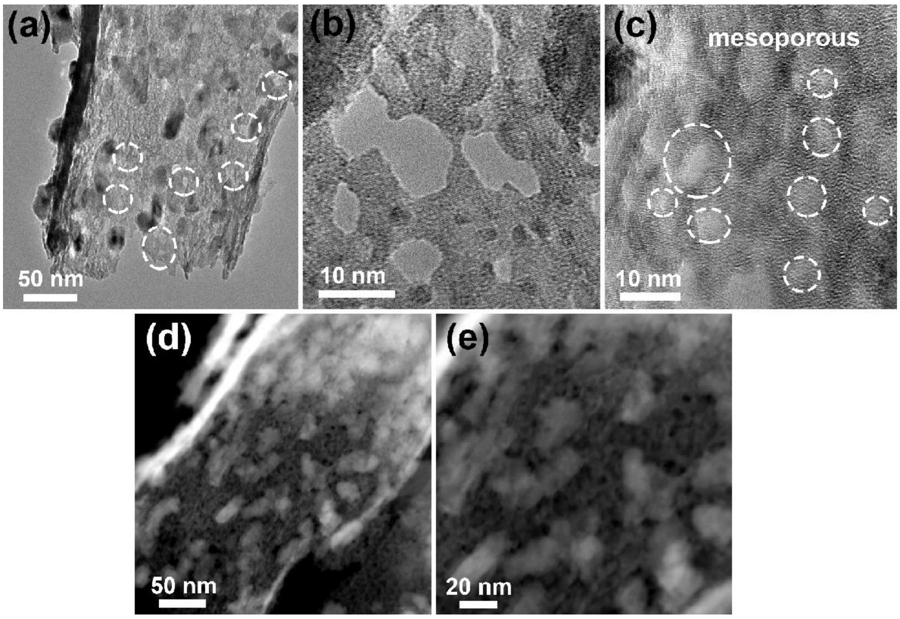

Fig. S16 (**a-e**) TEM, HRTEM, and HAADF-STEM images of NiCo@C-NiCoMoO/NF after HER stability test

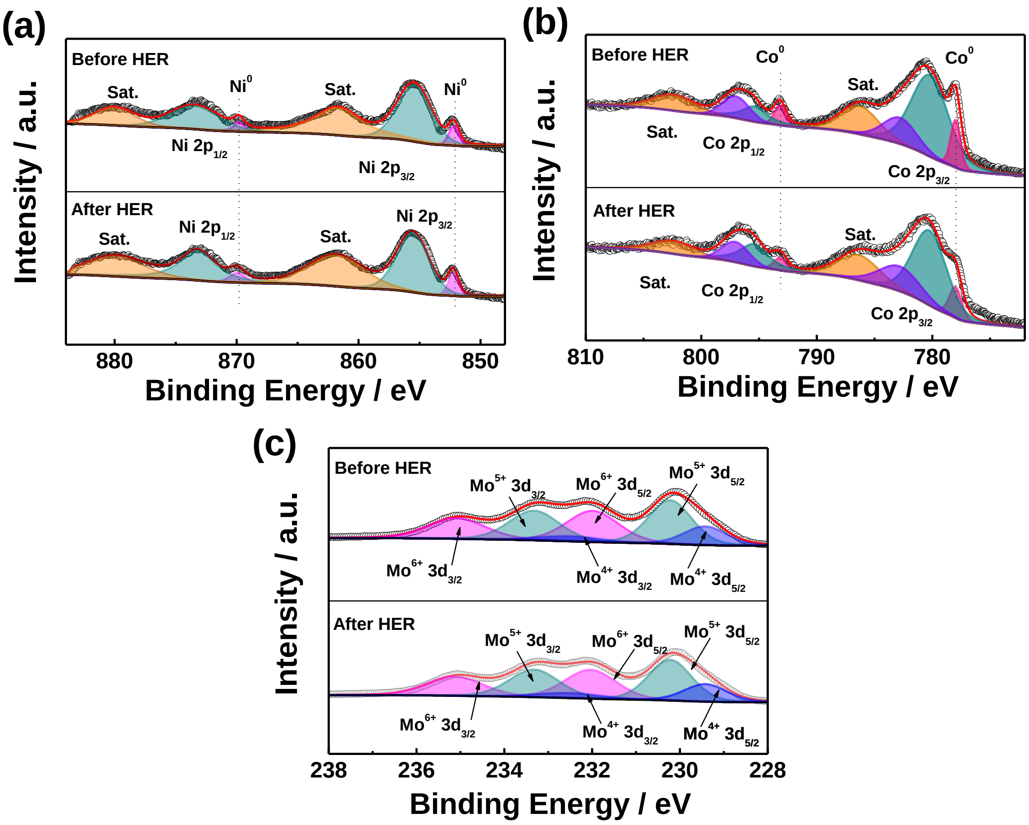

Fig. S17 (**a-c**) XPS spectra of NiCo@C-NiCoMoO/NF after HER stability test

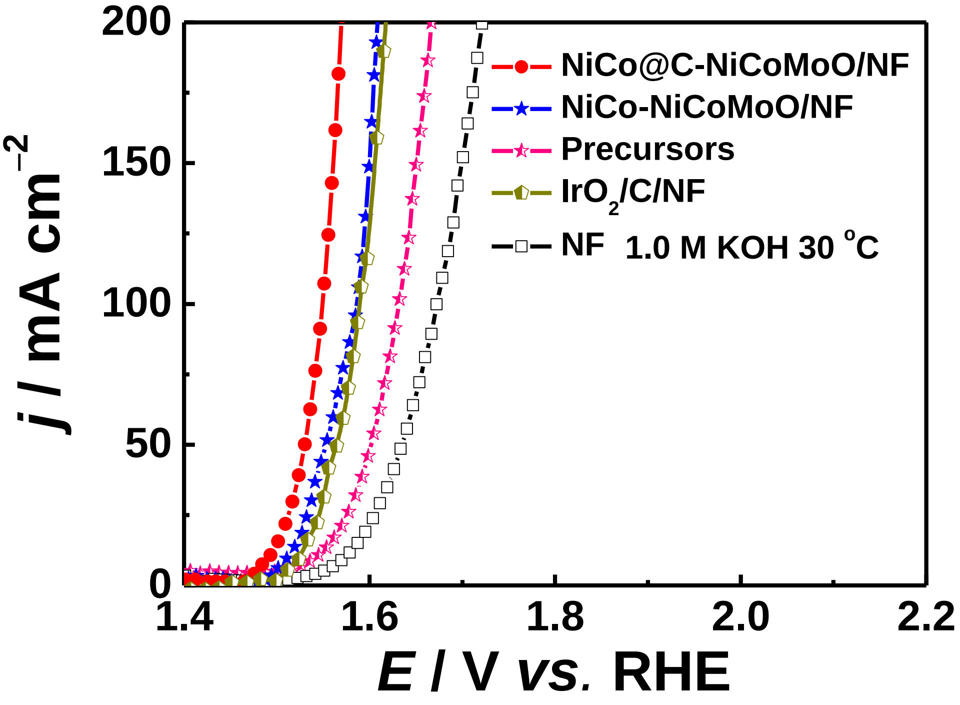

Fig. S18 LSV curves of OER for the investigated samples

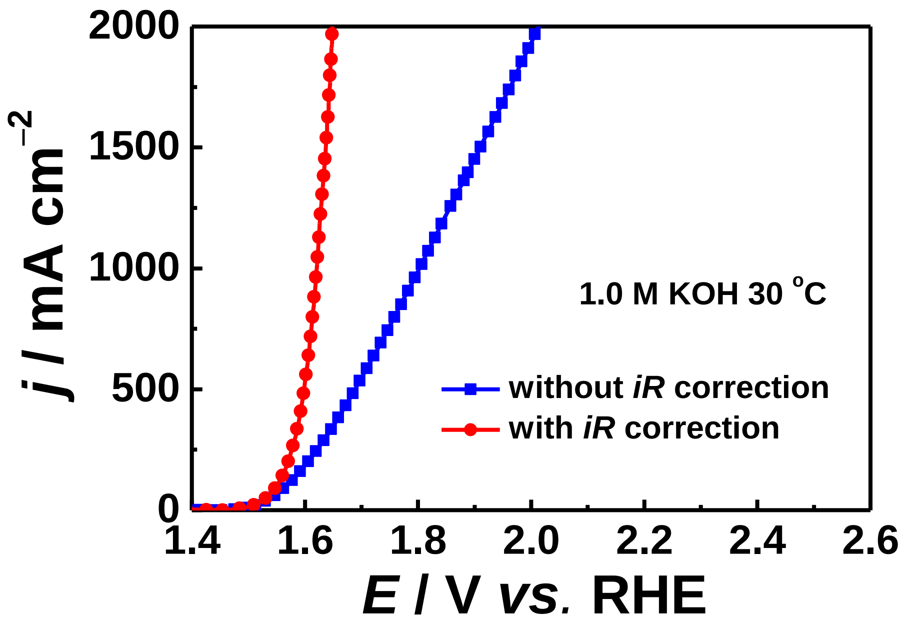

Fig. S19 LSV of NiCo@C-NiCoMoO/NF with/without *iR* correction

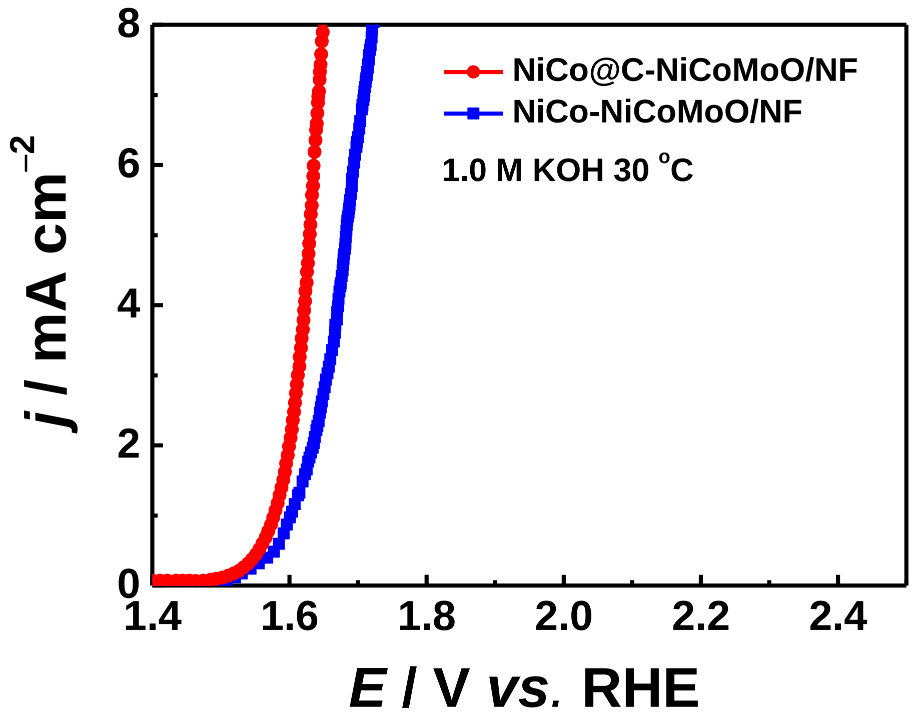

Fig. S20 LSV curves of OER for NiCo@C-NiCoMoO/NF and NiCo-NiCoMoO/NF normalized by EASAs

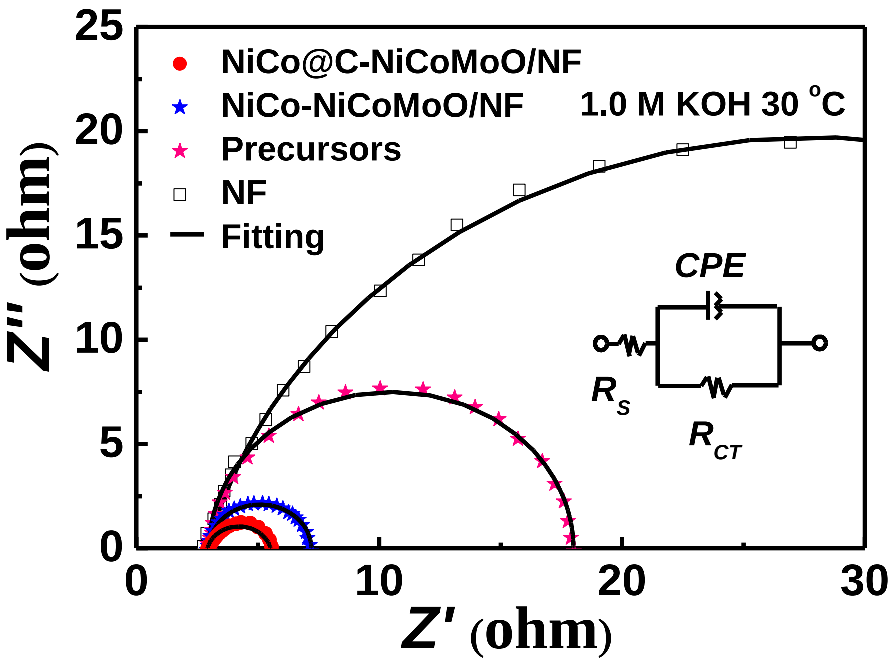

Fig. S21 Nyquist plots tested at 1.5 V for OER with a frequency from 100,000 to 0.1 Hz in 1.0 M KOH; inset is the equivalent circuit model

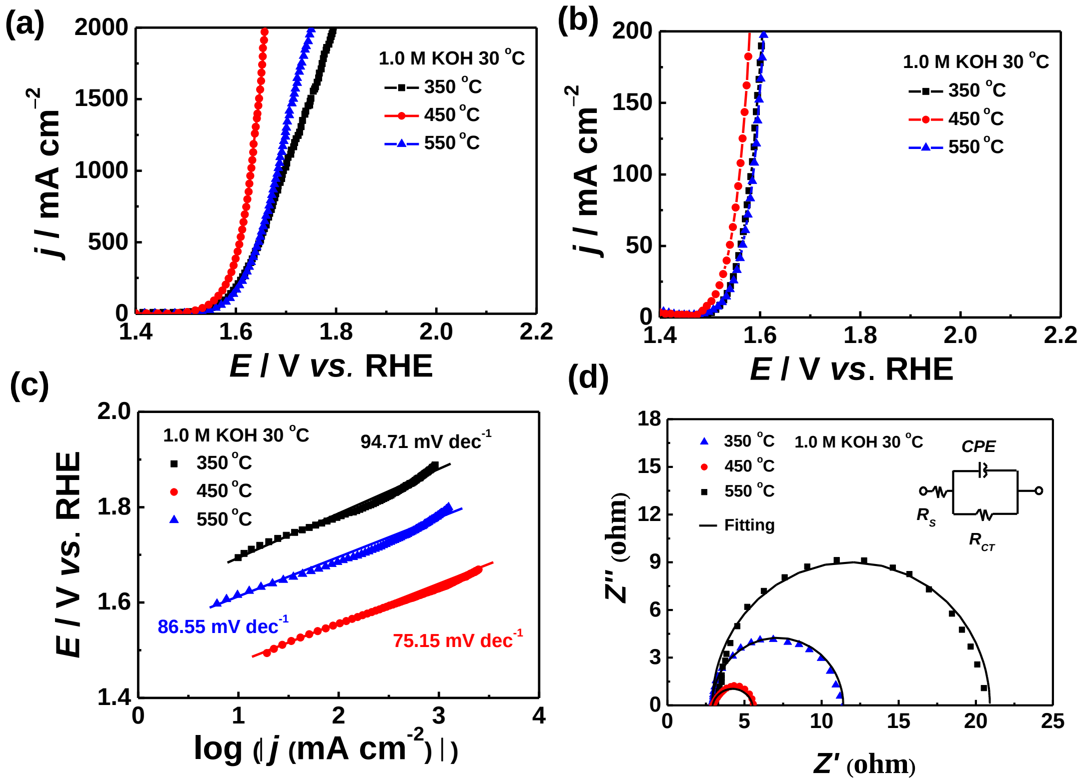

Fig. S22 (**a-d**) LSV curves, Tafel, and EIS of OER for precursors annealed at different temperatures

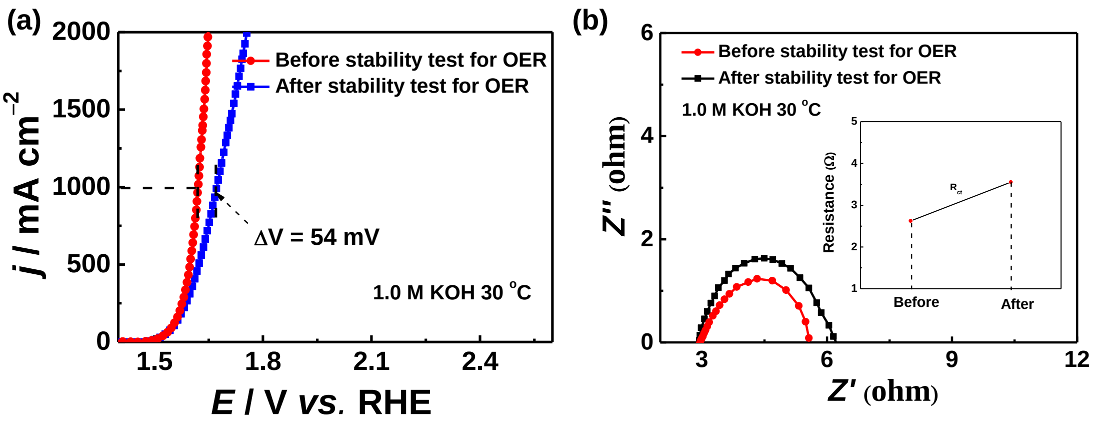

Fig. S23 (**a**) LSV curves and (**b**) R_{ct} of NiCo@C-NiCoMoO/NF before and after OER stability test

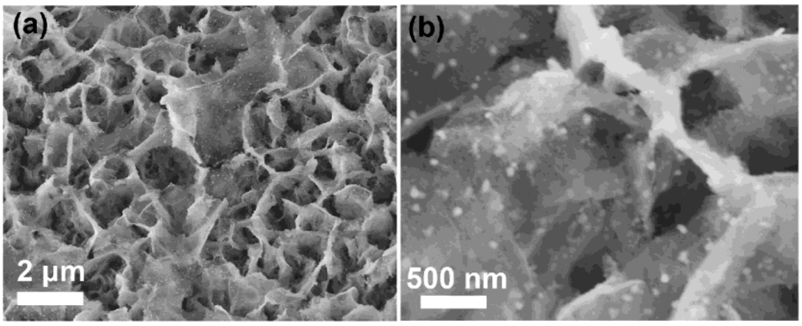

Fig. S24 SEM images of NiCo@C-NiCoMoO/NF after OER stability test

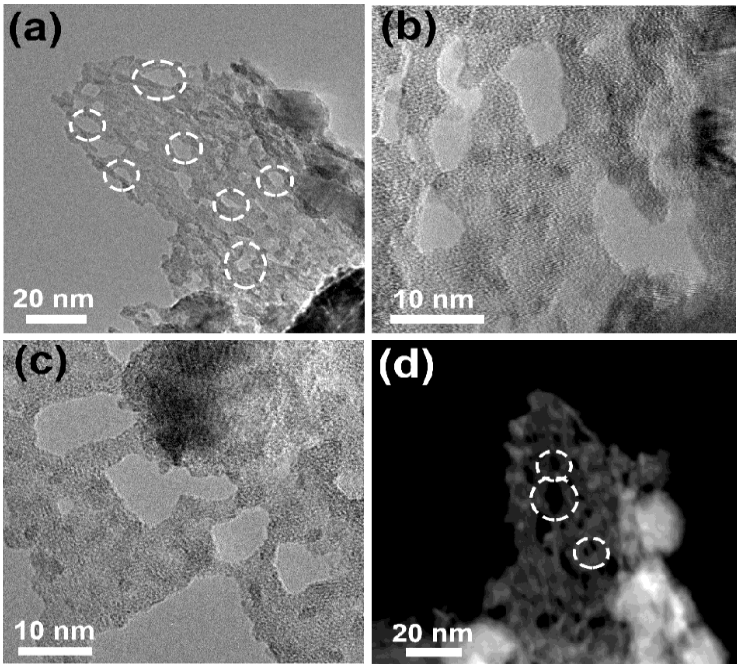

Fig. S25 (**a-d**) TEM, HRTEM, and HAADF-STEM images of NiCo@C-NiCoMoO/NF after OER stability test

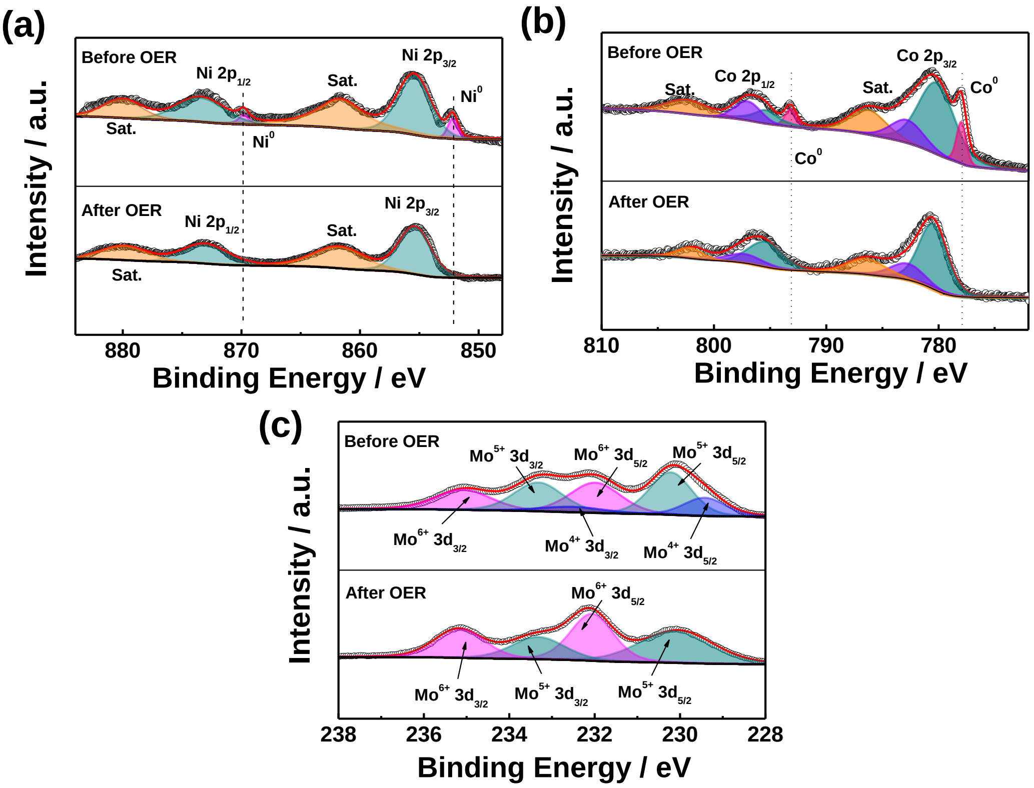

Fig. S26 (**a-c**) XPS spectra of NiCo@C-NiCoMoO/NF after OER stability test

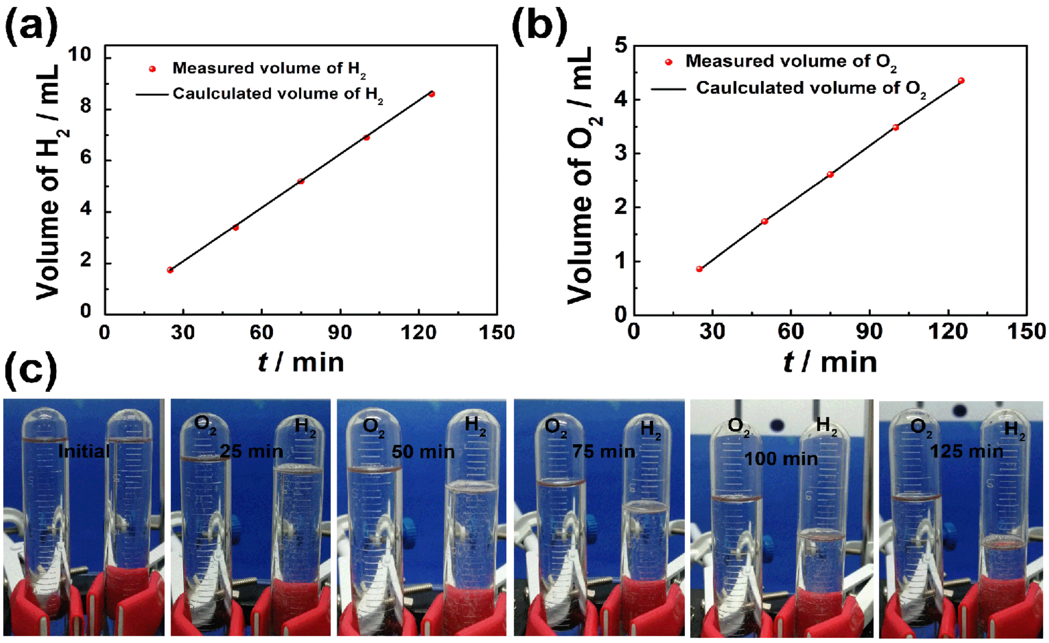

Fig. S27 (**a, b**) Volume of H_2 and O_2 theoretically calculated and actually measured at ±10.0 mA versus time for NiCo@C-NiCoMoO/NF in 1.0 M KOH solution; (**c**) volume of H_2 and O_2 at 0, 25, 50, 75, 100, and 125 min

Table S1 BET results of precursors annealed at different temperatures

**Catalysts**	**BET surface areas (m^2 g^{-1})**	**Pore volume (cm^3 g^{-1})**	**Pore size (nm)**
350 °C	58.69	0.23	15.36
450 °C	102.96	0.18	6.83
550 °C	37.36	0.25	27.05

Table S2 Elemental composition of NiCo@C-NiCoMoO/NF obtained from ICP-MS

**Element / wt%**	**Ni**	**Co**	**Mo**
NiCo@C-NiCoMoO/NF	31	15	22

Table S3 TOF and MA of NiCo@C-NiCoMoO/NF obtained at different overpotentials for HER

**Overpotentials (mV)**	**TOF (s^{-1})**	**MA (mA g^{-1})**
50	1.0×10^{-3}	2.6×10^{3}
100	4.0×10^{-3}	1.2×10^{4}
150	9.0×10^{-3}	2.7×10^{4}
200	2.0×10^{-2}	5.6×10^{4}

Table S4 Comparison TOF and MA of NiCo@C-NiCoMoO/NF for HER with other reported non-noble-metal catalysts

**Catalysts**	**TOF (s^{-1}@ mV)**	**MA (mA g^{-1}@ mV)**	**References**
NiCo@C-NiCoMoO/NF	4.0×10^{-3}@100	1.2×10^{4}@100	This work
$NiSe_2$-$CoSe_2$/NCF	2.13×10^{-3}@100	1.92×10^{3}@100	[S1]
$Ni_{12}P_5$-$Ni_4Nb_5P_4$/PCC	5.32×10^{-2}@100	3.05×10^{3}@100	[S2]
NiSe	7.5×10^{-1}@250	N/A	[S3]
Ni_3S_2-FeS-CoS/PNFCF	1.4×10^{-1}@100	N/A	[S4]
Cr-doped FeNi-P/NCN	2.14×10^{-1}@190	N/A	[S5]
Holey NCP	7.32×10^{-1}@200	N/A	[S6]
$Ni_{1.8}Cu_{0.2}$-P/NF	1.2×10^{-3}@100	N/A	[S7]
N-NiCoP/NCF	4.958×10^{-2}@200	N/A	[S8]
$NiSe_2$-$FeSe_2$	1.6×10^{-3}@300	5.53×10^{2}@100	[S9]
Mo-W-P/CC	1.9×10^{-2}@100	N/A	[S10]
N-NiVFeP/NFF	3.867×10^{-2}@180	N/A	[S11]
N-$NiCoP_x$/NCF	8.7×10^{-4}@40	3.14×10^{3}@100	[S12]
$MoSe_2$-$NiSe_2$-$CoSe_2$/PNCF	1.5×10^{-4}@80	1.38×10^{2}@100	[S13]
CoP UPNSs	N/A	1.51×10^{5}@100	[S14]
2H-MoS_2	N/A	~1.6×10^{4}@100	[S15]
2H-NbS_2	N/A	4.315×10^{4}@250	[S16]
NiS/Ni_2P/CC	9.01×10^{-1}@200	~5.8×10^{1}@200	[S17]
$NiCo_2P_x$	2.6×10^{-2}@100	N/A	[S18]

Table S5 TOF and MA of NiCo@C-NiCoMoO/NF obtained at different overpotentials for OER

Overpotentials (mV)	TOF (s^{-1})	MA ($mA\ g^{-1}$)
250	4.0×10^{-4}	3.0×10^{3}
300	2.7×10^{-3}	9.5×10^{3}
350	1.5×10^{-2}	4.5×10^{4}

Table S6 Comparison TOF and MA of NiCo@C-NiCoMoO/NF for OER with other reported non-noble-metal catalysts

Catalysts	TOF (s^{-1}@ mV)	MA ($mA\ g^{-1}$@ mV)	References
NiCo@C-NiCoMoO/NF	2.7×10^{-3}@350	9.5×10^{3}@300	This work
$NiSe_2$-$CoSe_2$/NCF	4.55×10^{-1}@300	5.28×10^{2}@300	[S1]
NiSe	~3×10^{-1}@320	N/A	[S3]
Amorphous NiFe-OH/NiFeP	3.6×10^{-2}@250	~5.0×10^{3}@250	[S19]
Ni_3S_2-FeS-CoS/PNFCF	1.2×10^{-3}@180	N/A	[S4]
Cr-doped FeNi-P/NCN	1.06×10^{-1}@140	N/A	[S5]
N-NiCoP/NCF	1.47×10^{-3}@200	N/A	[S8]
$NiSe_2$-$FeSe_2$	1.09×10^{-4}@300	1.338×10^{2}@100	[S9]
N-$NiCoP_x$/NCF	1.2×10^{-4}@300	1.014×10^{3}@400	[S12]
NiS/Ni_2P/CC	3.31×10^{-1}@300	~6.0×10^{1}@320	[S17]

Supplementary References

[S1] D.L. Chen, Z.M. Xu, W. Chen, G.L. Chen, J. Huang et al., Just add water to split water: Ultrahigh-performance bifunctional electrocatalysts fabricated using eco-friendly heterointerfacing of NiCo diselenides. J. Mater. Chem. A **8**, 12035-12044 (2020). https://doi.org/10.1039/D0TA02121K

[S2] D.L. Chen, Z.M. Xu, W. Chen, G.L. Chen, J. Huang et al., Mulberry-inspired nickel-niobium phosphide on plasma-defect-engineered carbon support for high-performance hydrogen evolution. Small **16**, 2004843 (2020). https://doi.org/10.1002/smll.202004843

[S3] H. Wu, X. Lu, G.F. Zheng, G.W. Ho, Topotactic engineering of ultrathin 2D nonlayered nickel selenides for full water electrolysis. Adv. Energy Mater. **8**, 1702704 (2018). https://doi.org/10.1002/aenm.201702704

[S4] Q. Zhang, W. Chen, G.L. Chen, J. Huang, C.S. Song, et al., Bi-metallic nitroxide nanodot-decorated tri-metallic sulphide nanosheets by on-electrode plasma-hydrothermal sprouting for overall water splitting. Appl. Catal. B **261**, 118254 (2020). https://doi.org/10.1016/j.apcatb.2019.118254

[S5] Y.Q. Wu, X. Tao, Y. Qing, H. Xu, F. Yang et al., Cr-doped FeNi-P nanoparticles encapsulated into N-doped carbon nanotube as a robust bifunctional catalyst for efficient overall water splitting. Adv. Mater. **31**, 1900178 (2019).

https://doi.org/10.1002/adma.201900178

[S6] Z.W. Fang, L.L. Peng, Y.M. Qian, X. Zhang, Y.J. Xie et al., Dual tuning of Ni-Co-A (A = P, Se, O) nanosheets by anion substitution and holey engineering for efficient hydrogen evolution. J. Am. Chem. Soc. **140**, 5241-5247 (2018). https://doi.org/10.1021/jacs.8b01548

[S7] S.J. Chu, W. Chen, G.L. Chen, J. Huang, R. Zhang et al., Holey Ni-Cu phosphide nanosheets as a highly efficient and stable electrocatalyst for hydrogen evolution. Appl. Catal. B **243**, 537-545 (2019). https://doi.org/10.1016/j.apcatb.2018.10.063

[S8] R. Zhang, J. Huang, G.L. Chen, W. Chen, C.S. Song et al., In situ engineering bi-metallic phospho-nitride bi-functional electrocatalysts for overall water splitting. Appl. Catal. B **254**, 414-423 (2019). https://doi.org/10.1016/j.apcatb.2019.04.089

[S9] J. Huang, S.T. Wen, G.L. Chen, W. Chen, G.X. Wang et al., Multiphase Ni-Fe-selenide nanosheets for highly-efficient and ultra-stable water electrolysis. Appl. Catal. B **277**, 119220 (2020). https://doi.org/10.1016/j.apcatb.2020.119220

[S10] X.D. Wang, Y.F. Xu, H.S. Rao, W.J. Xu, H.Y. Chen et al., Novel porous molybdenum tungsten phosphide hybrid nanosheets on carbon cloth for efficient hydrogen evolution. Energy Environ. Sci. **9**, 1468-1475 (2016). https://doi.org/10.1039/C5EE03801D

[S11] H.F. Fan, W. Chen, G.L. Chen, J. Huang, C.S. Song et al., Plasma-heteroatom-doped Ni-V-Fe trimetallic phospho-nitride as high-performance bifunctional electrocatalyst. Appl. Catal. B **268**, 118440 (2020). https://doi.org/10.1016/j.apcatb.2019.118440

[S12] R.X. Jin, J. Huang, G.L. Chen, W. Chen, B. Ouyang et al., Water-sprouted, plasma-enhanced Ni-Co phospho-nitride nanosheets boost electrocatalytic hydrogen and oxygen evolution. Chem. Eng. J. **402**, 126257 (2020). https://doi.org/10.1016/j.cej.2020.126257

[S13] G.X. Wang, W. Chen, G.L. Chen, J. Huang, C.S. Song et al., Trimetallic Mo-Ni-Co selenides nanorod electrocatalysts for highly-efficient and ultra-stable hydrogen evolution. Nano Energy **71**, 104637 (2020). https://doi.org/10.1016/j.nanoen.2020.104637

[S14] C. Zhang, Y. Huang, Y.F. Yu, J.F. Zhang, S.F. Zhuo et al., Sub-1.1 nm ultrathin porous CoP nanosheets with dominant reactive {200} facets: a high mass activity and efficient electrocatalyst for the hydrogen evolution reaction. Chem. Sci. **8**, 2769-2775 (2017). https://doi.org/10.1039/C6SC05687C

[S15] J. Kibsgaard, Z. Chen, B.N. Reinecke, T.F. Jaramillo, Engineering the surface structure of MoS_2 to preferentially expose active edge sites for electrocatalysis.

Nat. Mater. **11**, 963-969 (2012). https://doi.org/10.1038/nmat3439

[S16] J.C. Si, Q. Zheng, H.L. Chen, C.J. Lei, Y.G. Suo et al., Scalable production of few-Layer niobium disulfide nanosheets via electrochemical exfoliation for energy-efficient hydrogen evolution reaction. ACS Appl. Mater. Interfaces **11**, 13205-13213 (2019). https://doi.org/10.1021/acsami.8b22052

[S17] X. Xiao, D.K. Huang, Y.Q. Fu, M. Wen, X. Jiang et al., Engineering NiS/Ni_2P heterostructures for efficient electrocatalytic water splitting. ACS Appl. Mater. Interfaces **10**, 4689-4696 (2018). https://doi.org/10.1021/acsami.7b16430

[S18] R. Zhang, X.X. Wang, S.J. Yu, T. Wen, X.W. Zhu et al., Ternary $NiCo_2P_x$ nanowires as pH-universal electrocatalysts for highly efficient hydrogen evolution reaction. Adv. Mater. **29**, 1605502 (2017). https://doi.org/10.1002/adma.201605502

[S19] H.F. Liang, A.N. Gandi, C. Xia, M.N. Hedhili, D.H. Anjum et al., Amorphous NiFe-OH/NiFeP electrocatalyst fabricated at low temperature for water oxidation applications. ACS Energy Lett. **2**, 1035-1042 (2017). https://doi.org/10.1021/acsenergylett.7b00206
